# Impact of Gait Events Identification through Wearable Inertial Sensors on Clinical Gait Analysis of Children with Idiopathic Toe Walking

**DOI:** 10.3390/mi14020277

**Published:** 2023-01-21

**Authors:** Paolo Brasiliano, Guido Mascia, Paolo Di Feo, Eugenio Di Stanislao, Martina Alvini, Giuseppe Vannozzi, Valentina Camomilla

**Affiliations:** 1 Department of Movement, Human and Health Sciences, University of Rome “Foro Italico”, Piazza Lauro De Bosis 6, 00135 Rome, Italy; 2 Interuniversity Centre of Bioengineering of the Human Neuromusculoskeletal System, University of Rome “Foro Italico”, 00135 Rome, Italy; 3 “ITOP SpA Officine Ortopediche”, Via Prenestina Nuova 307/A, 00036 Palestrina, Italy

**Keywords:** ITW, MIMU, MEMS, ankle, pediatric gait

## Abstract

Idiopathic toe walking (ITW) is a gait deviation characterized by forefoot contact with the ground and excessive ankle plantarflexion over the entire gait cycle observed in otherwise-typical developing children. The clinical evaluation of ITW is usually performed using optoelectronic systems analyzing the sagittal component of ankle kinematics and kinetics. However, in standardized laboratory contexts, these children can adopt a typical walking pattern instead of a toe walk, thus hindering the laboratory-based clinical evaluation. With these premises, measuring gait in a more ecological environment may be crucial in this population. As a first step towards adopting wearable clinical protocols embedding magneto-inertial sensors and pressure insoles, this study analyzed the performance of three algorithms for gait events identification based on shank and/or foot sensors. Foot strike and foot off were estimated from gait measurements taken from children with ITW walking barefoot and while wearing a foot orthosis. Although no single algorithm stands out as best from all perspectives, preferable algorithms were devised for event identification, temporal parameters estimate and heel and forefoot rocker identification, depending on the barefoot/shoed condition. Errors more often led to an erroneous characterization of the heel rocker, especially in shoed condition. The ITW gait specificity may cause errors in the identification of the foot strike which, in turn, influences the characterization of the heel rocker and, therefore, of the pathologic ITW behavior.

## 1. Introduction

**Figure 1:**
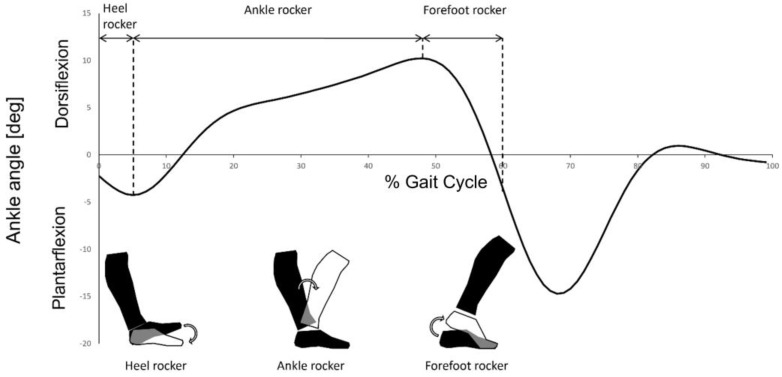
Graphical representation of the heel, ankle, and forefoot rockers within the stance phase (0–60% of the gait cycle). The heel rocker corresponds to the plantarflexion movement of the ankle at the beginning of the stance phase. The ankle rocker corresponds to the dorsiflexion movement during the most part of the stance phase. The forefoot rocker corresponds to the plantarflexion movement at the end of the stance phase.

Toe walking is a condition characterized by forefoot contact with the ground, normally present in the first developmental stage of walking [1]. It is also a common feature of certain pathologies (e.g., autism spectrum disorders, cerebral palsy) [2]. The diagnosis of idiopathic toe walking (ITW) is made by exclusion; the condition is defined as the presence of toe walking in healthy children older than three years of age with unknown causes. ITW tends to resolve naturally with time [2,3,4]; nevertheless, it has been hypothesized that it could lead to Achilles tendon shortening or other health issues (e.g., psychological discomfort for the children and their parents) if left untreated [5,6]. The need for a treatment and the selection of an optimal one should be based on an objective evaluation of the condition. In the scientific literature, the gait of children with ITW has been quantitatively described either in terms of number of strides with a toe walking pattern or in terms of the severity of ITW. The former classification distinguishes between typical or toe strides based on the vertical and forward acceleration of the foot [7]. The latter distinguishes between three severity levels based on both kinematics and kinetics features of the ankle joint [8]. Kinematics and kinetics data are obtained through instrumented gait analysis performed with optoelectronic systems and force platforms [8]. ITW severity classification is based on the characterization of three features: the ankle plantarflexion pattern at the beginning of the gait cycle (i.e., presence or absence of a heel rocker) [9] (Figure 1); the dorsiflexion pattern during the stance phase (i.e., timing of the forefoot rocker) [9] (Figure 1); the ankle kinetic profile (i.e., a predominant plantarflexion moment in the first part of the stance phase).

Although there are no clear and univocal guidelines for ITW treatment [10], therapeutical approaches focusing on the increase of ankle dorsiflexion excursion are clinically adopted. Treatments change according to the condition severity and child’s age [11]. According to the severity of the case, therapies range from surgery to non-invasive approaches [12]. Among these, there is the use of foot orthoses which limit ITW behaviour. They introduce mechanical constraints which induce the child to adopt a more physiological gait [13,14,15]. Treatment efficacy may be objectively quantified using optoelectronic systems, thus helping physicians in the process of choosing and tailoring it [16]. However, clinicians often report that children with ITW, if felt under examination, may adopt a physiological heel-to-toe gait pattern during gait analysis [17], thus compromising the test. Although a lab-based acquisition offers higher accuracy, a more ecological environment could help overcome this issue.

Ecological gait assessment can be facilitated by wearable measurement systems (WMSs) for estimating ankle mechanics. Thanks to the development of MEMS technology, Magneto-Inertial Measurement Units (MIMUs), composed of three mono- or tri-axial accelerometer, gyroscope, and magnetometer, allow the measurement of lower limb joint kinematics; pressure sensors, providing the force orthogonal to the sensor surface, can be used to estimate joint kinetics. WMSs have been widely used in many clinical contexts [18,19], allowing ambulatory gait analysis as well as a child’s gait monitoring during daily activities. Furthermore, they could be easily integrated with foot orthoses used for ITW treatment. When limiting the analysis to the kinematic component, the proximal and distal body segments orientation can be obtained from MIMU sensors through sensor fusion [20]. At the same time, their rigid transformation onto appropriate anatomical coordinate systems must be defined through anatomical calibration [21,22]. This kinematics estimation requires selecting:

(i) The foot modelling approach, which influences the potential of gait analysis to highlight deviations specific of ITW behaviour. Modelling the foot as a multisegment rigid body and tracking the talocrural behaviour from the rearfoot seems a preferable approach [23];

(ii) The anatomical calibration procedure. The relevant accuracy and reliability, validated in healthy individuals [21,24,25], should be tested in this population, and the most accurate and reliable should be chosen;

(iii) The anatomical coordinate systems (ACS) defined to obtain foot and shank orientation. ACSs should match the anatomical description commonly used in clinical settings [26] while granting for an adequate operator repeatability and operator and instrumental reliability. 

The determination of gait events delimiting the gait cycle (stride segmentation) is complementary but not secondary to the joint kinematics assessment. The identification of foot strike (FS) is critical since the severity classification of ITW relies on assessing ankle angular excursion at specific time instants or intervals of the gait cycle—GC—delimited by two subsequent FSs and then normalized to 100 points. Precisely: (i) the heel rocker (i.e., first rocker) is defined as an angle at FS greater than −5 deg, with a down going pattern in the first 12% GC; (ii) a premature forefoot rocker (i.e., third rocker) is defined as a peak dorsiflexion angle being present in the first 30% GC. Given the rapid change of the ankle angular excursion that occurs at the beginning of the GC (during loading response), even an error of a few samples could lead to an incorrect estimation of the ankle angle at FS and thus to a wrong identification of the presence/absence of the first rocker. Several MIMU-based algorithms for gait events detection have been validated in different clinical populations [27,28,29]. The reported studies were mainly based on foot and/or shank sensors [27,28,29] in line with the setup required to estimate ankle joint kinematics. Although most studies hypothesized that the proposed methods would work on pathological gait, less than one third were validated on such data [27] and only two considered a pediatric population presenting toe walking [30,31]. To date, no studies focusing on gait events identification using a wearable system in children with ITW are present in the scientific literature. 

The main purpose of this study is to evaluate the accuracy and precision of a selection of MIMU-based algorithms for foot strike estimation in children with ITW. Secondarily, the influence of the relevant errors on ITW severity classification [8] is evaluated. The algorithms were selected from the literature: two shank-based algorithms, chosen with the hypothesis that they could be less affected by a nontypical movement of the foot; one foot-based algorithm, chosen for its robustness in identifying both heel and forefoot contacts regardless of the foot movement pattern. The algorithms were tested on children with ITW walking either barefoot or while wearing a foot orthosis and tested with both marker-based stereophotogrammetry and MIMUs.

## 2. Materials and Methods

### 2.1. Participants

**Figure 2:**
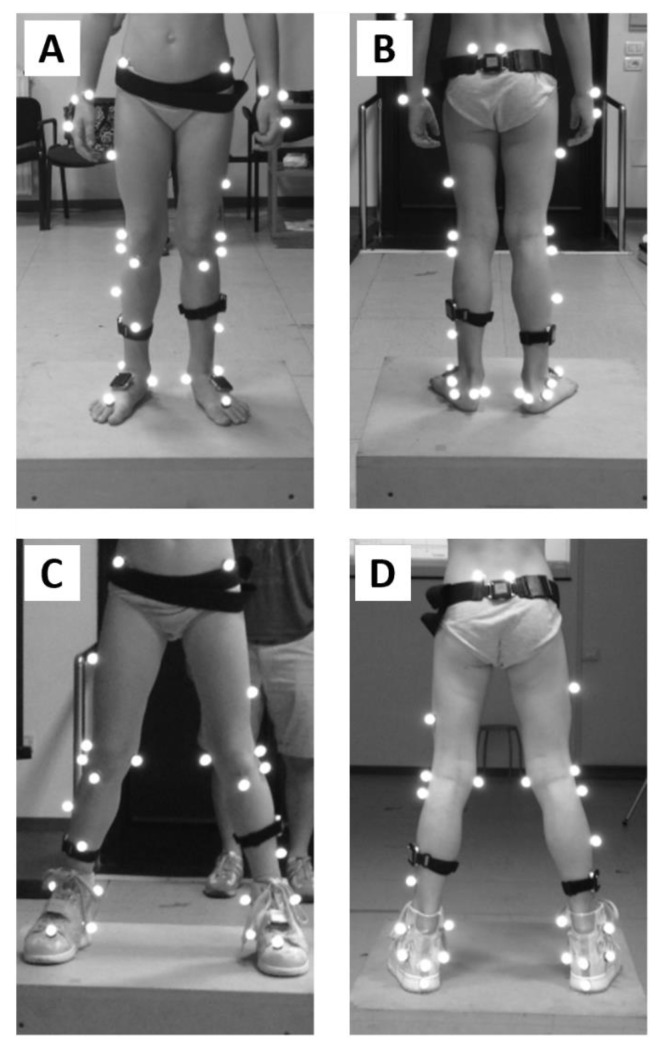
Markers and MIMU sensors positioning. Only a subset of the depicted markers was used in this study to implement the Plug-in-gait protocol. The MIMU sensor positioned on the pelvis was not used to perform any of the analysis reported here. (**A**,**B**): Front and rear views of the barefoot condition; (**C**,**D**): Front and rear views of the shoed condition.

Six children with ITW (4F, 2M; age 6.7 ± 2.2 y.o., stature 1.20 ± 0.12 m, body mass 24 ± 6 kg) were enrolled after an informed consent was signed by their parents. The study was approved by the institutional review board (University of Rome “Foro Italico”, Rome, Italy, CAR130/2022). Participants were included in the study after being diagnosed with ITW; thus, children with any pathologies that may cause toe walking and children younger than 3 years of age were excluded. The diagnosis of ITW and the prescription of a foot orthosis (Antiequinus Dynamic Orthosis—A.Dyn.O^®^, ITOP Officine Ortopediche, Italy; Figure 2C,D) were made by physiatrists of the Bambino Gesù Hospital in Rome. Physiatrists did not report limitation of ankle passive range of motion in any child considered in the study.

### 2.2. Experimental Procedure

Children underwent instrumented gait analysis walking at self-selected speed in a barefoot condition and while wearing the foot orthosis. Two operators, which engaged the children with games comprising the walking task, were positioned at the extremities of the walkway to avoid normalization of the gait pattern [17].

A seven infrared camera motion capture system (MTx, Vicon, Oxford, UK, sampling rate: 100 samples/s) was used to measure gait kinematics and to segment the gait cycle. A total of 14 markers were placed on the child for static and dynamic trials according to the Plug-in Gait lower-body model [32] (Figure 2). The textile design of the shoe used in combination with the orthosis allowed for palpation of anatomical landmarks, except for the one on the heel. This marker was identified as the most posterior point of the posterior profile of the shoe. 

A set of four MIMUs (OPAL, APDM wearable technologies, Portland, USA, accelerometer, and gyroscope full scale ranges: ±6 g, ±1500 deg/s, sampling rate: 128 samples/s) was also worn by the participants on shank and foot (Figure 2) to segment the gait cycle. They were selected among those available in the laboratory following the checklist by Hughes at al. [33]:

(i) Sensors’ mass and dimensions were small enough to minimise its influence (encumbrance) on the child’s movement;

(ii) Sensors were positioned to be in line with the articles presenting the MIMU-based algorithms. Precisely, shanks MIMUs were fixed on the lateral inferior portion of the segments compatibly with the marker-set and the foot orthosis used (Figure 2). Feet MIMUs were positioned on the dorsum of the foot (or over the shoes). To limit their wobbling and to avoid introducing movement artefacts due to fixing devices crossing the joint, MIMUs were fixed using bi-adhesive tape. Moreover, straps were used to prevent sensor fall in case of tape detachment. Sensor placement fixation was checked regularly during testing;

(iii) Sampling frequency was appropriate for the measured movement and outcome parameters (according to the Nyquist–Shannon sampling theorem and frequency analysis performed by Wu et al. [34]);

(iv) Sensor capacity (e.g., linear acceleration full-scale range) was appropriate to capture the true biomechanics of the movement of the distal segments (e.g., no clipping of the signal).

### 2.3. Data Analysis

#### 2.3.1. Lab-Based Gait Events Identification

**Figure 3:**
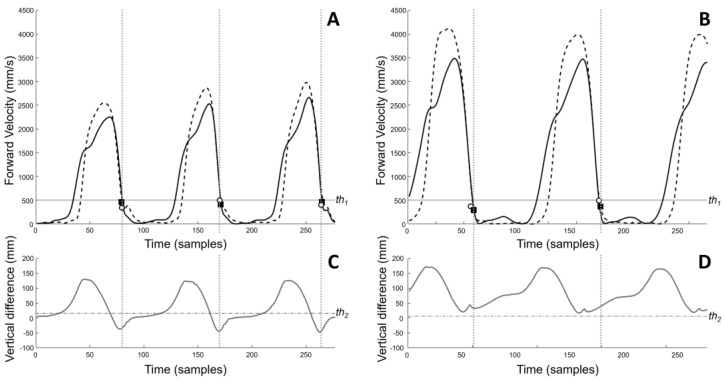
Top (**A**,**B**): Forward velocity of HEE (continuous thick black line) and TOE (dashed thick black line) for two trials representative of heel (left) and toe strike (right); horizontal thin continuous black line: th_1_. The white circles and black squares correspond to when the forward velocities of HEE and TOE fall below th_1_, respectively. Bottom (**C**,**D**): Difference of HEE and TOE vertical components (thick continuous grey line); horizontal thin dot−dashed grey line: th_2_. Depending on the value of this difference (below or above th_2_) a heel or toe FS was identified. For heel FS when HEE and TOE forward velocity fell below th_1_ (panel **A**), the difference between HEE and TOE vertical components was less than th_2_ (panel **C**); the HEE marker (white circles in panel **A**) was chosen as reference to identify FS_._ For toe FS when HEE and TOE forward velocity fell below th_1_ (panel **B**), the difference between HEE and TOE vertical components was greater than th_2_ (panel **D**); thus, the TOE marker (black squares in panel **B**) was chosen as reference to identify FS.

Foot strikes and foot offs (FO) were identified using stereophotogrammetric markers’ kinematic data. For FS, the algorithm proposed by Ghoussayni et al. [35] was used as described by Visscher et al. [36]. In their work [36], the authors examined the effect of using different markers to detect FSs in children walking normally, with foot flat, and on their toes. The authors found that the markers positioned on the heel (HEE) allowed for the best detection of a heel contact. Conversely, the markers placed on the second metatarsal head (TOE) were the best to detect forefoot contact. In the population enrolled in the current study, the contact type may change from stride to stride; therefore, the selection of the marker to be used (HEE or TOE) was based on the FS type (heel or forefoot, see Figure 3). To discriminate between heel and forefoot contact, the height difference (Z_diff_) of the vertical component of the two markers was considered:

Z_diff_(k) = HEE_z_(k) − TOE_z_(k), (1)

A threshold (th_2_) was set as the average height difference during a static trial:

th_2_ = Σ^N^_i=1_ Z_diff_(i)/N (2)

over the N samples recorded during this trial. 

During the walking trials, a threshold (th_1_) of 500 mm/s was set for forward velocity (V_F_) of HEE and TOE to detect a FS as the time sample k for which the following conditions were both satisfied:

V_F_(k) < th_1_ and V_F_(k − 1) > th_1_, (3)

where k and k − 1 are the current and previous time sample, respectively.

A heel or forefoot strike were, therefore, identified when the following conditions were satisfied: 

heel FS:

V_F_(k) < th_1_ and V_F_ (k − 1) > th_1_ and Z_diff_(k) < th_2_ (4)

forefoot FS:

V_F_ (k) < th_1_ and V_F_ (k − 1) > th_1_ and Z_diff_(k) > th_2_ (5)

Regarding FOs identification, the method proposed and validated by O’Connor et al. [37] was used; as in this work, no marker was placed on the hallux, which is the best position identified to estimate foot off by Visscher et al. [36]. Marker kinematic data were low-pass filtered using a different Butterworth filter for each gait-identified event (FS: 2nd order, cut-off frequency = 2 Hz [35]; FO: 4th order, cut-off frequency = 7 Hz [37]).

#### 2.3.2. MIMU-Based Gait Events Identification

**Figure 4:**
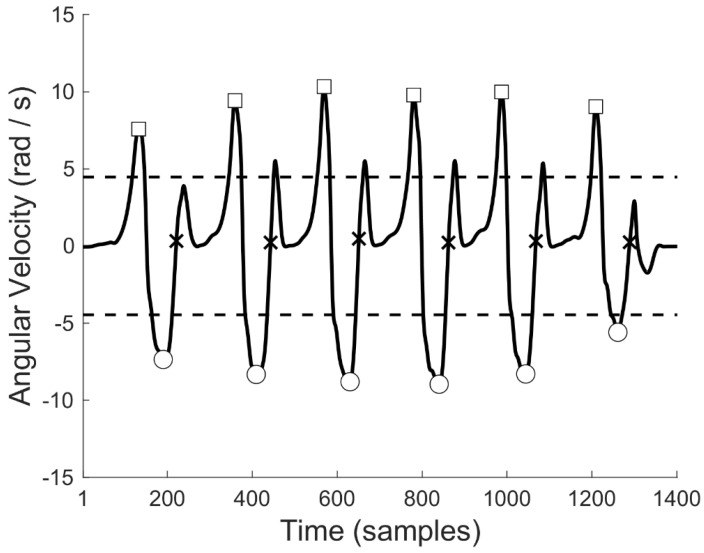
Medial-lateral angular velocity of the foot-mounted IMU for a linear gait representative of the population (continuous line); the circles are the local minima with a magnitude bigger than the signal standard deviation (below dashed line); FSs (crosses) are identified as the first ascending zero-crossing time sample after each of the local minima, roughly coinciding with the mid-swing phase of the gait cycle; FOs (squares) are the local maxima preceding the mid−swing local minima and bigger than the signal standard deviation (above dashed line).

Three MIMU-based algorithms were used to detect gait events (FSs and FOs) and to test whether it was better for this population to use a MIMU placed on the shank [38,39] or on the foot [34]. The two shank-based algorithms, named TRO [38] and SAL [39], were implemented as described in the original works. The foot-based algorithm proposed by Wu et al. [34] (thereinafter referred to as WUM), was slightly modified. This method, as several others in the literature [40], relies on the identification of local maxima of the foot’s medial-lateral angular velocity. However, when using it on current barefoot data, results were not reliable due to inconsistent medial-lateral angular velocity maximal excursion during gait strides. To limit this inconsistency, we relied only on the local minima, as they roughly correspond to the mid-swing phase of the gait cycle (Figure 4). First, local minima of the foot-mounted IMU angular velocity ω were identified as those time samples k satisfying the following condition:

ω(k) < ω(k − 1) and ω(k) < ω(k + 1) and ω(k) < std(ω)(6)

where k, k − 1, and k+1 are the current, previous, and subsequent sample, and std (ω) is the standard deviation of the signal. Furthermore, a minimal time interval of 0.5 s was required to identify two consecutive local minima. Once the local minima were identified (Figure 4), the sample of FS occurrence (k^FS^) was identified as the first sample k, occurring after the local minima, satisfying the following conditions:

ω(k^FS^) ⋅ ω(k^FS^ − 1) < 0 and ω(k^FS^) > ω(k^FS^ − 1) (7)

In a similar way, the sample of FO (k^FO^) was detected as the first sample k corresponding to a local maximum and occurring before the considered local minimum.

ω(k^FO^ − 1) < ω(k^FO^) and ω(k^FO^ + 1) < ω(k^FO^) and ω(k^FO^) > std(ω) (8)

Prior to each recording session, MIMUs were recalibrated and signals bias removed following the procedure described by Bergamini et al. [41]. To implement TRO and SAL algorithms, MIMUs signals were filtered using a low-pass filter (7th order Butterworth; cut-off frequency = 6.5 Hz). To implement the WUM algorithm, the instant of zero-crossing was identified on raw data; data were low-pass filtered (2nd order Butterworth; cut-off frequency = 5 Hz) to find local minima and maxima.

### 2.4. Characterization of Heel and Forefoot Rockers

Heel and forefoot rockers were characterized according to the definitions given in the ITW severity classification [8]. With the aim of excluding the errors related to MIMUs orientation estimation, ankle kinematics was obtained from marker data. Ankle kinematics was filtered using a low-pass filter (4th order Butterworth; cut-off frequency = 6 Hz). This marker-based kinematics was segmented into gait cycles according to FSs identified with MIMUs and used to characterize the abovementioned rockers. Reference gait temporal parameters (stride, stance and swing duration) were computed from lab-based gait events. MIMU-based gait events were used to obtain the same temporal parameters for comparison.

### 2.5. Statistical Analysis

Gait events identified with the three MIMU-based algorithms and gait temporal parameters were compared with reference data identified using the markers kinematic data. Algorithms’ accuracy and precision were quantified, respectively, in terms of bias and 95% limits of agreements (LoAs) calculated performing Bland–Altman analyses (lab-based parameters—MIMU-based parameters) [42]. The impact of the errors in FS and FO identification on the characterization of heel and forefoot rockers was evaluated through the identification of true positives (TP), true negatives (TN), false positives (FP), and false negatives (FN). Successively, we calculated:

true positive rate (TPR)

TPR = TP/(TP + FN)(9)

true negative rate (TNR)

TNR = TN/(TN + FP)(10)

false positive ratio (FPR)

FPR = FP/(FP + TN)(11)

false negative ratio (FNR)

FNR = FN/(FN + TP)(12)

The choice of the algorithms was driven by the criterion of having the highest TPR and TNR and the lowest FPR and FNR.

## 3. Results

**Figure 5:**
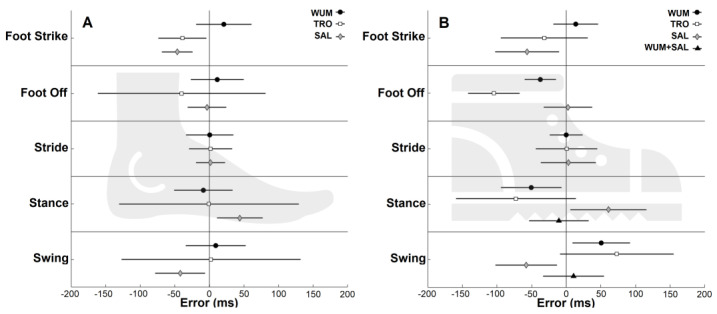
Biases of WUM (black circle), TRO (white square), and SAL (grey diamond) with limits of agreements (horizontal black lines) for gait events identification and gait temporal parameters: (**A**) barefoot condition (left) and (**B**) shoed condition (right). To optimize stance/swing estimation, mixed solutions were also tested. The combination improving results is reported (biases are represented as black triangles) for stance and swing duration in a shoed condition. These were estimated identifying FSs with WUM and FOs with SAL.

Results for all the algorithms in both conditions are summarized in Figure 5. 

In the barefoot condition, the most accurate and precise algorithms for FSs identification were WUM (bias: 21 ms) and SAL (LoAs: 22 ms), respectively. For FOs identification, SAL was the most accurate and precise algorithm (bias: −3 ms; LoAs: 28 ms). For all the gait temporal parameters estimation, the most precise algorithm was SAL (LoAs: 21 ms, 33 ms, 36 ms for stride, stance, and swing duration, respectively). The most accurate algorithm for stride duration estimation was WUM, with null bias. For stance and swing duration estimation, the most accurate algorithm was TRO (bias: −1 ms and 2 ms, respectively). 

In the shoed condition, FSs were most accurately and precisely estimated with WUM (bias: 14 ms; LoAs: 32 ms). For FOs identification, the most accurate and precise were SAL (bias: 2 ms) and WUM (LoAs: 23 ms), respectively. For all the gait temporal parameters, WUM was the most accurate and precise algorithm (bias: null, −51 ms, 50 ms; LoAs: 24 ms, 44 ms, 42 ms for stride, stance, and swing duration, respectively). Given the high biases for stance and swing duration estimation, WUM and SAL were used in combination, the former for FSs identification and the latter for FOs identification. With this combination the results for both stance and swing duration estimation improved (bias: −11 ms, 11 ms for stance and swing duration, respectively).

**Figure 6:**
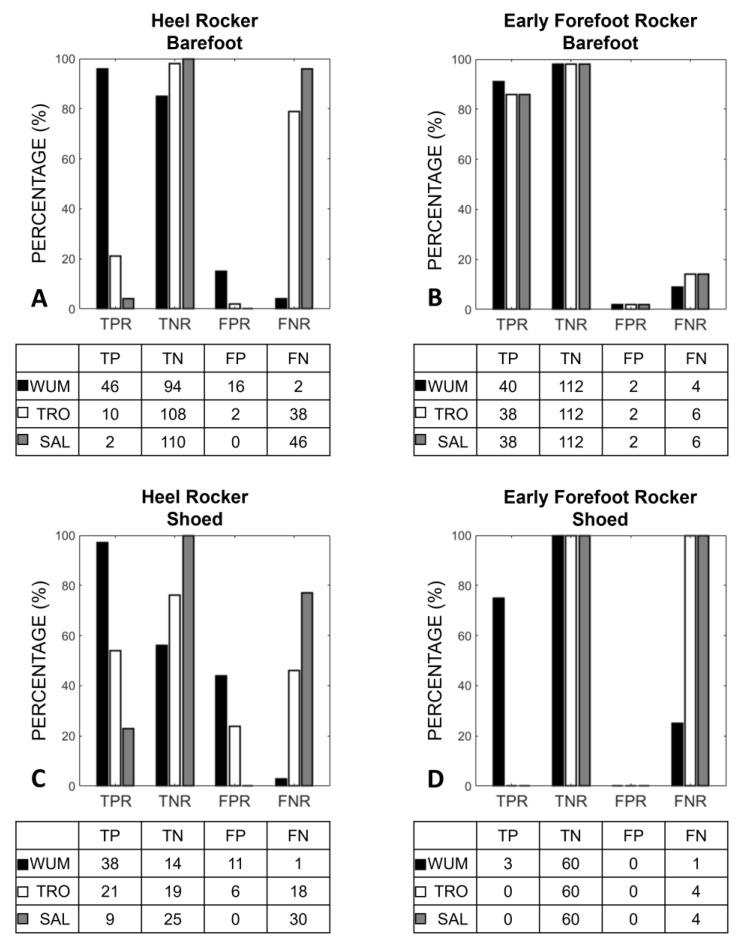
Characterization of heel rocker during barefoot (**A**) and shoed (**C**) walking and early forefoot rocker during barefoot (**B**) and shoed (**D**) walking, based on the FSs identified with the three algorithms. In the graphs, black, white, and grey bars represent WUM, TRO, and SAL, respectively.

The characterization of the heel and forefoot rockers are graphically represented in terms of true and false positive and negative rates (Figure 6). For the first rocker in the barefoot condition, WUM was the algorithm with the highest TPR (96%) and the lowest FNR (2%), while SAL was the algorithm with the highest TNR (100%) and the lowest FPR (0%). Concerning the characterization of the forefoot rocker in a barefoot condition, the best algorithm was WUM with TPR, TNR, FPR, and FNR of 91%, 98%, 2%, and 9%, respectively. 

In the shoed condition, for both heel and forefoot rockers characterization, the highest TPR (97%) and TNR (100%) were achieved using WUM and SAL, respectively, while the lowest FPR (0%) and FNR (3%) were achieved using SAL and WUM, respectively.

## 4. Discussion

This study compared the performance of three MIMU-based algorithms in identifying gait events in children with ITW walking barefoot and while wearing a foot orthosis. Although no single algorithm emerges as the best one from all perspectives, a selection of preferable algorithms for event identification, temporal parameters estimation and ITW classification was devised, depending on the barefoot/shoed condition. 

Concerning gait events identification, FSs and FOs were best identified by different algorithms: FSs identification had the smallest bias when performed with the WUM algorithm, while FOs were identified more accurately via SAL algorithm. Regarding gait temporal parameters, when calculating stride duration, only the bias of FSs identification affects the duration, with errors similar for all algorithms and conditions (about 2 ms). On the contrary, stance and swing duration are affected by the combination of the biases of FSs and FOs identification. In a barefoot condition, TRO tends to identify both FSs and FOs with similar delayed biases, while the other algorithms have different biases for FSs and FOs. Consequently, stance and swing duration have the smallest bias when using the TRO algorithm (−0.6 and 2.4 ms, compared to WUM −9 and 9 ms, and to SAL, 44 and −42 ms, respectively). Nevertheless, complementing bias information with the LoA values (±120 and ±45 ms for TRO and WUM, respectively) WUM appears to be the best algorithm to estimate gait temporal parameters in a barefoot condition. In the shoed condition, all the algorithms presented average biases of FSs and FOs that differs within each other in terms of magnitude and/or sign. Therefore, the biases for stance and swing duration were higher compared to barefoot walking. These biases can be reduced by combining two algorithms with similar biases of FSs and FOs identification. The best combination uses WUM for FSs and SAL for FOs (Figure 5B), resulting also in the best LoA (43 ms for stance and swing phases duration). 

Regarding the identification of foot rockers, the forefoot rocker was almost always correctly identified, both when normal (2 FP) and when anticipated (1–4 FN). Conversely, none of the analyzed algorithms performed clearly better than the other for the characterization of the heel rocker. This is not surprising since even a small error in the identification of the initial contact can lead to a misidentification of the presence of a heel rocker (e.g., the WUM average biases of 21 ms and 14 ms represent the 2% and 1.4% of the mean stride duration and correspond to a 15% and 44% FPR in the barefoot and shoed condition, respectively). A heel rocker is present if the planta-dorsiflexion angle is over −5 deg at FS. Indeed, even a slightly anticipated or delayed FS (the highest bias being 5 samples for SAL in the shoed condition) can cause an important difference in the estimation of this angle at foot strike. In children with ITW, the plantar-flexion movement can occur either in the terminal swing phase or during the loading response phase, leading to toe or heel strikes, respectively. For a toe strike, the identification of an FS prior to the real event could lead to identifying a nonexistent heel rocker. For a heel strike, the identification of an FS after the real event could lead to denying a real heel rocker. The WUM algorithm overestimated the presence of a heel rocker, while underestimating its absence in both conditions. On the contrary, TRO and SAL underestimated the presence of the heel rocker, still underestimating its absence. Overestimating the presence of heel strikes is to be avoided at a diagnosis stage, whereas it is important to early identify a child as a toe walker. Conversely, underestimating it could lead to considering an effective rehabilitation intervention as not effective. Clinicians could choose if dealing with a higher false positive or negative rate for heel rocker identification according to the clinical need; alternatively, the evaluation could be performed with different algorithms and be integrated with a video analysis in case of a discrepancy.

The study must be interpreted considering the following limitations. First, to increase the number of identified gait events, reference data was obtained from marker trajectories and not using force platforms. The accuracy of the marker-based approach has been assessed in the order of 1 and 6 ms for children with a toe walking and a typical walking pattern, respectively. Such discrepancies are unlikely to affect the results of this study since differences between marker-based and MIMU-based events were in the range 14–21 ms. Second, the analysis of how an anticipated or delayed identification of FSs would influence the characterization of the heel and forefoot rocker must be interpreted with caution. In the current study, to set apart the errors entailed in estimating ankle kinematics with MIMUs [24] from those relative to gait event identification, ankle kinematics was estimated using an optoelectronic system. Therefore, the characterization of the heel and forefoot rocker may change when using MIMUs to estimate the ankle kinematics. 

Future studies shall develop a wearable measurement system to perform instrumented gait analysis in an ecological environment. A step forward from the segmentation of the gait cycle to the assessment of ankle kinematics using MIMUs is required. Such a system would be capable of correctly revealing the typical walking patterns of this population that may be hindered using lab-based optical motion capture. Lastly, wearable systems could inform clinicians on the severity of the condition by monitoring its development, hence, proving the efficacy of eventual treatments if prescribed.

## 5. Conclusions

The present study found that among the analyzed algorithms, WUM shall be used every time only FSs are used in the analysis (FS identification and stride duration), while SAL is indicated for FOs identification and for computing stance and swing duration. Although relatively small compared to the whole gait cycle duration, the errors induced by the MIMU-based algorithms for foot strike estimation led to an erroneous characterization of the heel rocker, especially in the shoed condition. Nevertheless, the WUM algorithm allowed a promising characterization of heel and forefoot rockers, provided that its tendency to overestimate the presence of heel rockers is taken in due account.

## Data Availability

The data presented in this study are available on request from the corresponding authors. The data are not publicly available due to privacy restrictions.
